# Development and validation of a predictive model for the risk of possible sarcopenia in middle-aged and older adult diabetes mellitus in China

**DOI:** 10.3389/fpubh.2025.1521736

**Published:** 2025-04-03

**Authors:** Mengyuan Qiao, Haiyan Wang, Mengzhen Qin, Taohong Xing, Yingyang Li

**Affiliations:** ^1^School of Nursing, Henan University of Science and Technology, Luoyang, China; ^2^Xinjiang Emergency Center, People’s Hospital of Xinjiang Uygur Autonomous Region, Ürümqi, China; ^3^Endoscopy Centre, The First Affiliated Hospital of Henan University of Science and Technology, Luoyang, China; ^4^Department of Critical Care Surgery, The First Affiliated Hospital of Henan University of Science and Technology, Luoyang, China

**Keywords:** diabetes mellitus, middle-aged and older adults, sarcopenia, prediction model, nomogram

## Abstract

**Background:**

People with diabetes mellitus (DM) have a significantly increased risk of sarcopenia. A cross-sectional analysis was performed using nationally representative data to evaluate possible sarcopenia in middle-aged and older adults with diabetes mellitus, and to develop and validate a prediction model suitable for possible sarcopenia in middle-aged and older adults with diabetes mellitus in the Chinese community.

**Methods:**

Data from the China Health and Retirement Longitudinal Study (CHARLS), which focuses on people 45 years of age or older, served as the basis for the prediction model. CHARLS 2015 participants were used in the study, which examined 53 factors. In order to guarantee model reliability, the study participants were split into two groups at random: 70% for training and 30% for validation. Ten-fold cross-validation and Least Absolute Shrinkage and Selection Operator (LASSO) regression analyses were used to determine the best predictors for the model. The factors associated with sarcopenia in DM were researched using logistic regression models. Nomogram were constructed to develop the predictive model. The performance of the model was assessed using area under the curve (*AUC*), calibration curves and decision curve analysis (*DCA*).

**Results:**

A total of 2,131 participants from the CHARLS database collected in 2015 passed the final analysis, and the prevalence of sarcopenia was 28.9% (616/2131). Eight factors were subsequently chosen as predictive models by LASSO logistic regression: age, residence, body mass index, diastolic blood pressure, cognitive function, activities of daily living, peak expiratory flow and hemoglobin. These factors were used in the nomogram predictive model, which showed good accuracy and agreement. The *AUC* values for the training and validation sets were 0.867 (95%CI: 0.847~0.887) and 0.849 (95%CI: 0.816~0.883). Calibration curves and DCA indicated that the nomogram model exhibited good predictive performance.

**Conclusion:**

The nomogram predictive model constructed in this study can be used to evaluate the probability of sarcopenia in middle-aged and older adult DM, which is helpful for early identification and intervention of high-risk groups.

## 1 Introduction

Diabetes mellitus (DM) is a metabolic disease that is hyperglycemia caused by defective insulin secretion or action. The prevalence of DM is high, with one in eleven people worldwide currently diagnosed with DM (1). According to the statistics of the International Diabetes Federation (IDF), as of 2021, the global DM patients have reached 537 million cases, of which 6.3 million died of DM, and it is expected that by 2045, the global DM patients will reach nearly 784 million, of which China leads the world in diabetes cases with 140 million diabetics in 2021 and has the fastest growing diabetes incidence in the world (2). Notably, the prevalence is highest in the middle-aged and older adult population (3). With the progression of DM, a series of complications occur when the function of organs such as kidneys, blood vessels, nervous system and eyes of the body are affected (1). In recent years, sarcopenia has been recognized as the third type of complication in diabetic patients, presenting a reversible character (4).

The European Working Group on Sarcopenia (EWGSOP) was the first to publish a consensus on sarcopenia, defining sarcopenia as a geriatric syndrome with loss of muscle mass, loss of muscle strength and/or reduced somatic function associated with ageing (5). It has been reported that the number of patients with muscle wasting disorders will reach 200 million worldwide in 2050 (6). As muscle mass declines, it increases the risk of falls, disability, re-hospitalisation and death in patients, leading to an increase in adverse clinical events and placing a significant strain and economic burden on healthcare systems and society (7).

DM and sarcopenia are mutually reinforcing, they share common risk factors and age-related trends (5). Therapeutic strategies for DM limit energy intake, accelerating the loss of muscle strength and muscle mass in patients, further increasing the incidence of sarcopenia (8). The risk of sarcopenia in diabetic mellitus patients is 1.5–3 times higher than that in the non-diabetic people (9, 10). Significantly, patients with sarcopenia also exacerbate glucose metabolism disorders due to their decreased muscle mass and function (11), and the two disorders contribute to each other.

The prevalence and related variables of sarcopenia in older persons are currently the subject of increased research, whereas fewer studies have developed risk prediction models for the risk of sarcopenia in DM. A predictive model for sarcopenia in the Chinese older adult population has been developed by based on the CHARLS database (12), but the study was conducted only in the older adult, and was not applicable to the specific population of middle-aged and older adult DM. To improve the prognosis of the DM population, it is crucial to construct a predictive model appropriate for the risk of sarcopenia in middle-aged and older DM patients.

## 2 Methods

### 2.1 Study participants

**Figure 1:**
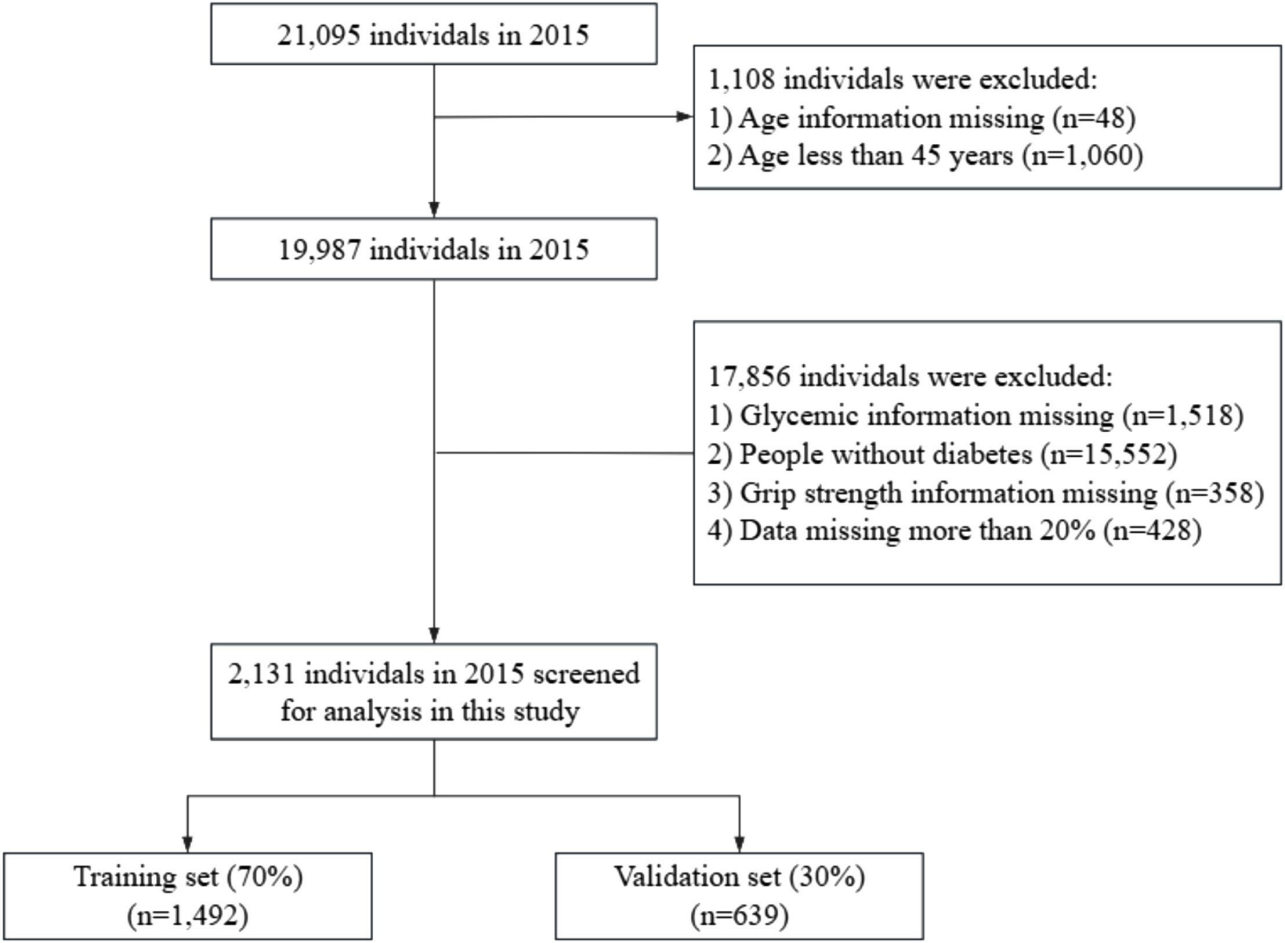
Flowchart of the study.

The data used for this article were obtained from the CHARLS investigators and are publicly available at http://charls.pku.edu.cn. The CHARLS is an ongoing longitudinal survey that provides high-quality microdata on households and individuals aged 45 years and older in China With the aim of analyzing population ageing issues and promoting interdisciplinary research on ageing. The project received approval from the Biomedical Ethics Committee (IRB00001052-11015) of Peking University in Beijing, China, and our study strictly adhered to the principles outlined in the Declaration of Helsinki; informed consent was obtained from all participants. For this study, we used data from the 2015 CHARLS to extract demographic background, general health status, disease history, and biochemical parameters. The inclusion criteria were as follows: (a) age ≥ 45 years; (b) having diabetes mellitus; (c) answering the questions about sarcopenia. Data missing by more than 20% were excluded. Finally, a total of 2,131 respondents participated in the study. The data filtering process is shown in Figure 1.

### 2.2 Assessment of diabetes

During blood collection for the CHARLS data, we asked participants to fast the night before. Blood collection was performed by medical professionals. If participants were unable to meet the fasting requirement, blood samples were still collected and blood glucose values were analyzed as higher random plasma glucose (RPG). Participants with incident DM were identified based on the following criteria (13): previously diagnosed with diabetes; higher hemoglobin A1c (HbA1c) level (≥6.5%); higher fasting plasma glucose (FPG) level (≥126 mg/dL); and/or RPG level (≥200 mg/dL).

### 2.3 Assessment of sarcopenia

In this study, we adopted the Asian Working Group for Sarcopenia (AWGS) 2019 standard to define and evaluate sarcopenia using three indexes: muscle strength, physical function and appendicular skeletal muscle mass (ASM) (14), specific assessment of sarcopenia can be found in Supplementary material.

#### 2.3.1 Muscle strength

Muscle strength was measured using a grip strength meter, and values below 28 kg for males and below 18 kg for females indicate decreased muscle strength.

#### 2.3.2 Physical function

Physical function included the gait speed, the five-time chair stand test, and the short physical performance battery (SPPB). The total score of SPPB was 12 points with 4 points for each test. According to the AWGS 2019 recommendations, low physical performance was defined as 6-m walking speed <1 m/s or 5-times chair stand test time ≥ 12 s, or SPPB score ≤ 9 points.

#### 2.3.3 Appendicular skeletal mass (ASM)

In our article, we used an anthropometric equation to estimate the muscle mass, which has previously been validated in Chinese individuals, and the ASM equation model showed a high level of agreement with DXA (15, 16).


$$ASM=0.193\times \text{weight}\;\left(\text{kg}\right)+0.107\times \text{height}\;\left(\text{cm}\right)–4.157\times \text{gender}\;\left(\text{male}=1,\text{female}=2\right)–0.037\times \text{age}–2.631$$

Height-adjusted muscle mass was calculated as ASM/Ht^2^ = ASM/height (m)^2^. The cut-off point for low muscle mass was based on the lowest 20% percentile of ASM/Ht^2^ in the study population. Since our data are derived from the 2015 CHARLS data, we refer to the criteria of Wu et al. (17). Therefore, the ASM/Ht^2^ cut-off for female was <5.08 kg/m^2^, and the ASM/Ht^2^ cut-off for male was <6.88 kg/m^2^.

### 2.4 Predictors

#### 2.4.1 Demographic characteristics

Demographic characteristics include age, body mass index, sex, marital status, residence, education, pension insurance, and health insurance.

#### 2.4.2 Vital signs

Vital signs including temperature, pulse, respiratory rate, systolic blood pressure (SBP) and diastolic blood pressure (DBP) at the first records after admission.

#### 2.4.3 Health status and behavior

Health status and behaviors include smoking, drinking, history of disease (chronic disease, hypertension, cancer, lung disease, heart disease, stroke, arthritis, dyslipidaemia, liver disease, kidney disease, stomach disease, asthma), fall down, sleep duration, myopia, hyperopia, hearing, self-reported health status, peak expiratory flow (PEF), activities of daily living (ADL) and social participation.

(1) The CHARLS assesses the respondents’ ability to perform activities of daily living using the Basic Activities of Daily Living (BADL) and Instrumental Activities of Daily Living (IADL), with six items selected for the BADL: dressing, bathing, eating, getting in and out of bed, going to the toilet, and controlling urine and faeces; and six items for the IADL: doing household chores, cooking, shopping on one’s own, making a phone call, taking medication, and controlling money. In both BADL and IADL, completing all 6 items without difficulty was considered to be functionally intact, and completing any one of them without difficulty was defined as functionally impaired (18, 19).
(2) The data on participation in social activities were obtained from the CHARLS questionnaire, ‘Did you do any of the following activities in the past month (multiple answers allowed)’, with the following answers: (a)visiting the home, socializing with friends; (b) playing mahjong, chess, cards, going to the community room; (c) providing help to your relatives, friends or neighbors who do not live with you, without any compensation, friends or neighbors; (d) going to parks or other places to dance, work out, practice qigong; (e) taking part in community organizations; (f) volunteering or charitable activities; (g) taking care of sick people or people with disabilities you do not live with free of charge; (h) going to school or attending training courses; (i) speculating on stocks (funds and other financial securities); (j) surfing the internet; (k) others; (l) none of the above. For the above 12 options, if you choose any one of (a) to (l), you are considered to have participated in activities, and it is marked as ‘1’, while if you choose (l), you are considered to have not participated in social activities, and it is marked as ‘0’ (20, 21).

#### 2.4.4 Mental health parameters

Mental health parameters included self-reported depression and cognitive function.

(1) Depression was assessed by the 10-item score of the Centre for Epidemiological Studies Depression Scale (CESD-10) from the CHARLS data. It was divided into 4 levels according to scores of 0, 1, 2, and 3, and the 10-item scores ranged from 0–30, with >10 defined as having a tendency to be depressed (22, 23).
(2) Measurement of cognitive function included two dimensions, situational memory and mental state, with overall cognitive function scores ranging from 0 to 21, with higher scores indicating better cognitive function. (a) Situational memory: a total score of 10 points, divided into instantaneous memory and delayed recall. Instantaneous memory was measured by asking the respondents to immediately recall the 10 words just read to them by the investigator; delayed recall required the respondents to recall the same 10 words again after 4 to 10 min. One point was awarded for each correctly recalled word, and the average of the two tests was taken as the total situational memory score. (b) Mental state: The total score is 11 points, divided into time orientation, calculation ability and drawing ability. Time orientation requires respondents to answer the day of the year, month, day, season and day of the week, answer 1 correctly scored 1 point, a total of 5 points. Computing ability requires respondents to carry out five calculations (answer 100 minus 7 equals how much, the answer to the value of the value of the answer and then subtracted from the 7, repeat 4 times), if the respondent calculations are wrong, but the results of the next calculation is equal to the last error value minus 7, can still be scored one point, a total of five points. Drawing ability requires respondents were asked to draw a picture of two overlapping pentagrams displayed by the investigator, and one point was awarded to those who drew the picture (24).

#### 2.4.5 Biochemical parameters

After the home interview, 8 mL of fasting venous blood was collected by professional staff and the samples were sent to the laboratory of the Chinese Center for Disease Control and Prevention (CDC) in Beijing, China, where they were stored at −80°C. Hemoglobin (Hb), white blood cell (WBC), c-reactive protein (CRP), Hematocrit (HCT), mean corpuscular volume (MCV), Platelets (PLT), total cholesterol (TC), triglyceride (TG), high-density lipoprotein cholesterol (HDL-c), low-density lipoprotein cholesterol (LDL-c), Blood Urea Nitrogen (BUN), Creatinine, Uric acid and Cystatin C were collected by a professional staff.

### 2.5 Statistical analyses

Statistical analyses were performed using R 4.3.0. The CHARLS data were randomly divided into a training set and an internal validation set in a ratio of 7:3.

(1) Statistical description: quantitative data conforming to normal distribution were expressed as means ± standard deviations, and the *t*-test was used for inter-group comparisons. Non-normally distributed data were expressed as medians and tertile range, and the rank sum test was used for inter-group comparisons.
(2) Predictor variable screening: model over-fitting was prevented by LASSO regression, penalties on coefficients prevented the problem of covariance, and logistic regression was subsequently performed to determine the independent risk factors for sarcopenia in middle-aged and older adult diabetic populations. All tests were two-sided, and *p* < 0.05 was considered a statistically significant difference.
(3) Nomogram predictive model construction: logistic regression was used to construct the predictive model, and the ‘rms’ package was used to construct the nomogram predictive model for sarcopenia.
(4) Evaluation of the model: the C_index of the sarcopenia nomogram predictive model was calculated to assess the discriminatory ability of the model, and the calibration curve of the sarcopenia predictive model was produced to determine the degree of agreement between the predicted probability and the observed results. Clinical validity was assessed by decision curve analysis (*DCA*).
(5) Model validation: *AUC*, calibration curves and *DCA* were used to validate the predictive performance of the nomogram model.

## 3 Results

### 3.1 Participants characteristics

Our study included 2,131 middle aged and older adult diabetic patients. The mean age was 63.32 ± 8.90 years, of which 1,227 (57.6%) were male and 904 (42.4%) were female. The prevalence of sarcopenia in middle-aged and older adult diabetic patients was 28.9% (616/2,131). Baseline characteristics are shown in Supplementary Table 1. Among middle-aged and older adult diabetic patients, 1,492 (70%) and 639 (30%) were randomly assigned to the training and validation sets, respectively. The results in Supplementary Table 2 show that there was no significant difference (*p* > 0.05) between the predictors of the training and validation date sets and the baselines were comparable. The baseline characteristics of the training set participants are shown in Table 1.

**Table 1:** Baseline characteristics of the training set participants.

Variables	Overall (*n* = 1,492)	No sarcopenia (*n* = 1,063)	Possible sarcopenia (*n* = 429)	*p*-value
Demographic characteristics				
Age, Median (IQR)	63.0 (57.00,69.00)	61.00 (55.00,67.00)	69.00 (62.00,75.00)	<0.001
BMI (kg/m^2^)	25.03 (22.62,27.65)	25.81 (23.96,28.28)	22.08 (19.72,24.89)	<0.001
Sex (%)				<0.001
Male	650 (43.6)	512 (48.2)	138 (32.2)	
Female	842 (56.4)	551 (51.8)	291 (67.8)	
Education (%)				<0.001
Primary school or below	704 (47.2)	434 (40.8)	270 (62.7)	
Junior high school/ technical secondary school	610 (40.9)	479 (45.1)	131 (30.5)	
High school and above	178 (11.9)	150 (14.1)	28 (6.5)	
Marital status (%)				<0.001
Married	1,258 (84.3)	938 (88.2)	320 (74.6)	
Unmarried	234 (15.7)	125 (11.8)	109 (25.4)	
Residence (%)				<0.001
Urban	622 (41.7)	470 (44.8)	146 (34.0)	
Rural	870 (58.3)	587 (55.2)	283 (66.0)	
Medical insurance (%)				0.016
No	108 (7.2)	66 (6.2)	42 (9.8)	
Yes	1,384 (92.8)	997 (93.8)	387 (90.2)	
Endowment insurance (%)				0.001
No	541 (36.3)	457 (43.0)	84 (19.6)	
Yes	951 (63.7)	606 (57.0)	345 (80.4)	
Vital signs				
Temperature (°C)	36.7 (36.6, 37.0)	36.7 (36.6, 37.0)	36.6 (36.5; 37.0)	0.615
Pulse (times/min)	75.50 (68.00,83.00)	75.00 (67.50,82.50)	76.50 (69.00,84.50)	0.014
Respiratory rate (times/min)	20.0 (20.0, 20.0)	20.0 (20.0, 20.0)	19.0 (19.0, 20.0)	0.471
SBP (mmHg)	130.00 (118.13,145.38)	130.00 (119.50,145.50)	130.50 (114.50,145.00)	0.276
DBP (mmHg)	75.50 (68.00,83.50)	76.50 (69.00,84.00)	71.50 (65.50,80.25)	<0.001
Health status and behavior				
Smoking (%)				<0.001
No	860 (57.6)	575 (54.1)	285 (66.4)	
Yes	632 (42.4)	488 (45.9)	144 (33.6)	
Drinking (%)				0.006
No	835 (56.0)	571 (53.7)	264 (61.5)	
Yes	657 (44.0)	492 (46.3)	165 (38.5)	
Chronic diseases (%)				0.670
No	129 (8.6)	94 (8.8)	35 (8.2)	
Yes	1,363 (91.4)	969 (91.2)	394 (91.8)	
Hypertension (%)				0.673
No	684 (45.8)	491 (46.2)	193 (45.0)	
Yes	808 (54.2)	572 (53.8)	236 (55.0)	
Cancer (%)				0.743
No	1,465 (98.2)	1,043 (98.1)	422 (98.4)	
Yes	27 (1.8)	20 (1.9)	7 (1.6)	
Chronic lung disease (%)				0.001
No	1,270 (85.1)	925 (87.0)	345 (80.4)	
Yes	222 (14.9)	138 (13.0)	84 (19.6)	
Heart disease (%)				0.173
No	1,077 (72.2)	778 (73.2)	299 (69.7)	
Yes	415 (27.8)	285 (26.8)	285 (26.8)	
Stroke (%)				0.012
No	1,397 (93.6)	1,006 (94.6)	391 (91.1)	
Yes	95 (6.4)	57 (5.4)	38 (8.9)	
Arthritis (%)				<0.001
No	787 (52.7)	607 (57.1)	180 (42.0)	
Yes	705 (47.3)	456 (42.9)	249 (58.0)	
Dyslipidemia (%)				<0.001
No	930 (62.3)	617 (58.0)	313 (73.0)	
Yes	562 (37.7)	446 (42.0)	116 (27.0)	
Liver disease (%)				0.347
No	1,350 (90.5)	957 (90.0)	393 (91.6)	
Yes	142 (9.5)	106 (10.0)	36 (8.4)	
Kidney disease (%)				0.290
No	1,311 (87.9)	928 (87.3)	383 (89.3)	
Yes	181 (12.1)	135 (12.7)	46 (10.7)	
Stomach disease (%)				0.019
No	1,016 (68.1)	743 (69.9)	273 (63.6)	
Yes	476 (31.9)	320 (30.1)	156 (36.4)	
Asthma (%)				<0.001
No	1,375 (92.2)	1,003 (94.4)	372 (86.7)	
Yes	117 (7.8)	60 (5.6)	57 (13.3)	
Fall down (%)				0.002
No	1,187 (79.6)	868 (81.7)	319 (74.4)	
Yes	305 (20.4)	195 (18.3)	110 (25.6)	
Tap water (%)				0.001
No	368 (24.7)	238 (22.4)	130 (30.3)	
Yes	1,124 (75.3)	825 (77.6)	299 (69.7)	
ADL (%)				<0.001
Non-disability	938 (62.9)	727 (68.4)	211 (49.2)	
Disability	554 (37.1)	336 (31.6)	218 (50.8)	
Social participation (%)				<0.001
No	664 (44.5)	423 (39.8)	241 (56.2)	
Yes	828 (55.5)	640 (60.2)	188 (43.8)	
Hyperopia (%)				<0.001
Good	409 (27.4)	248 (23.3)	161 (37.5)	
Fair	723 (48.5)	530 (49.9)	193 (45.0)	
Poor	360 (24.1)	285 (26.8)	75 (17.5)	
Myopia (%)				0.002
Good	354 (23.7)	229 (21.5)	125 (29.1)	
Fair	766 (51.3)	550 (51.7)	216 (50.3)	
Poor	372 (24.9)	284 (26.7)	88 (20.5)	
Hearing (%)				<0.001
Good	251 (16.8)	153 (14.4)	98 (22.8)	
Fair	798 (53.5)	580 (54.6)	218 (50.8)	
Poor	443 (29.7)	330 (31.0)	113 (26.3)	
Self-assessed health status (%)				<0.001
Good	507 (34.0)	321 (30.2)	186 (43.4)	
Fair	747 (50.1)	561 (52.8)	186 (43.4)	
Poor	238 (16.0)	181 (17.0)	57 (13.3)	
Sleep time (h)	6.00 (5.00,8.00)	6.00 (5.00,8.00)	6.00 (5.00,8.00)	0.297
PEF (L/min)	300.00 (220.00,380.00)	329.00 (250.00,400.00)	240.00 (170.00,310.00)	<0.001
Mental health parameters				
Depression (scores)	8.00 (4.00,13.00)	7.00 (3.00,12.00)	10.00 (5.00,16.00)	<0.001
Cognitive function (scores)	11.50 (8.50,14.00)	12.00 (9.50,14.50)	9.50 (6.50,12.35)	<0.001
Biochemical parameters				
Hb (g/dL)	13.70 (12.60,14.80)	13.90 (12.80,15.00)	13.00 (12.00,14.10)	<0.001
WBC (1,000)	6.10 (5.10,7.32)	6.13 (5.19,7.33)	5.84 (4.93,7.30)	0.021
CRP (mg/L)	2.00 (1.00,3.57)	2.10 (1.10,3.50)	1.80 (0.90,5.60)	0.084
HCT (%)	41.35 (38.00,44.60)	42.00 (38.70,45.10)	39.70 (36.45,43.25)	<0.001
MCV (fl)	91.20 (87.10,94.80)	91.20 (87.30,94.60)	91.60 (87.05,95.35)	0.289
PLT (10^9^/L)	203.00 (159.00,246.00)	204.00 (162.00,246.00)	201.00 (154.00,246.00)	0.295
TC (mg/dl)	187.64 (163.03,213.13)	187.64 (162.93,212.74)	186.87 (163.32,213.32)	0.879
TG (mg/dL)	143.36 (98.23,209.73)	150.44 (104.42,221.24)	122.12 (85.84,177.88)	<0.001
HDL-c (mg/dL)	47.49 (40.93,54.83)	46.33 (40.15,53.28)	49.81 (42.66,59.46)	<0.001
LDL-c (mg/dL)	102.32 (84.17,122.78)	103.09 (84.56,122.78)	101.16 (83.01,123.55)	0.666
BUN (mg/dL)	15.13 (12.61,18.21)	15.13 (12.61,18.21)	15.13 (12.61,18.49)	0.823
Creatinine (mg/dL)	0.74 (0.63,0.89)	0.75 (0.64,0.88)	0.72 (0.63,0.90)	0.489
Uric acid (mg/dL)	4.90 (4.10,6.00)	5.00 (4.20,6.00)	4.90 (3.90,5.80)	0.025
Cystatin C (mg/L)	0.86 (0.74,0.98)	0.83 (0.72,0.95)	0.93 (0.78,1.06)	<0.001

BMI, body mass index; SBP, systolic blood pressure; DBP, diastolic blood pressure; ADL, activities of daily living; PEF, peak expiratory flow; Hb, hemoglobin; WBC, white blood cell; CRP, c-reactive protein; HCT, hematocrit; MCV, mean corpuscular volume; PLT, platelets; TC, total cholesterol; TG, triglyceride; HDL-c, high-density lipoprotein cholesterol; LDL-c, low-density lipoprotein cholesterol; BUN, blood urea nitrogen.

### 3.2 LASSO and logistic regression

**Figure 2:**
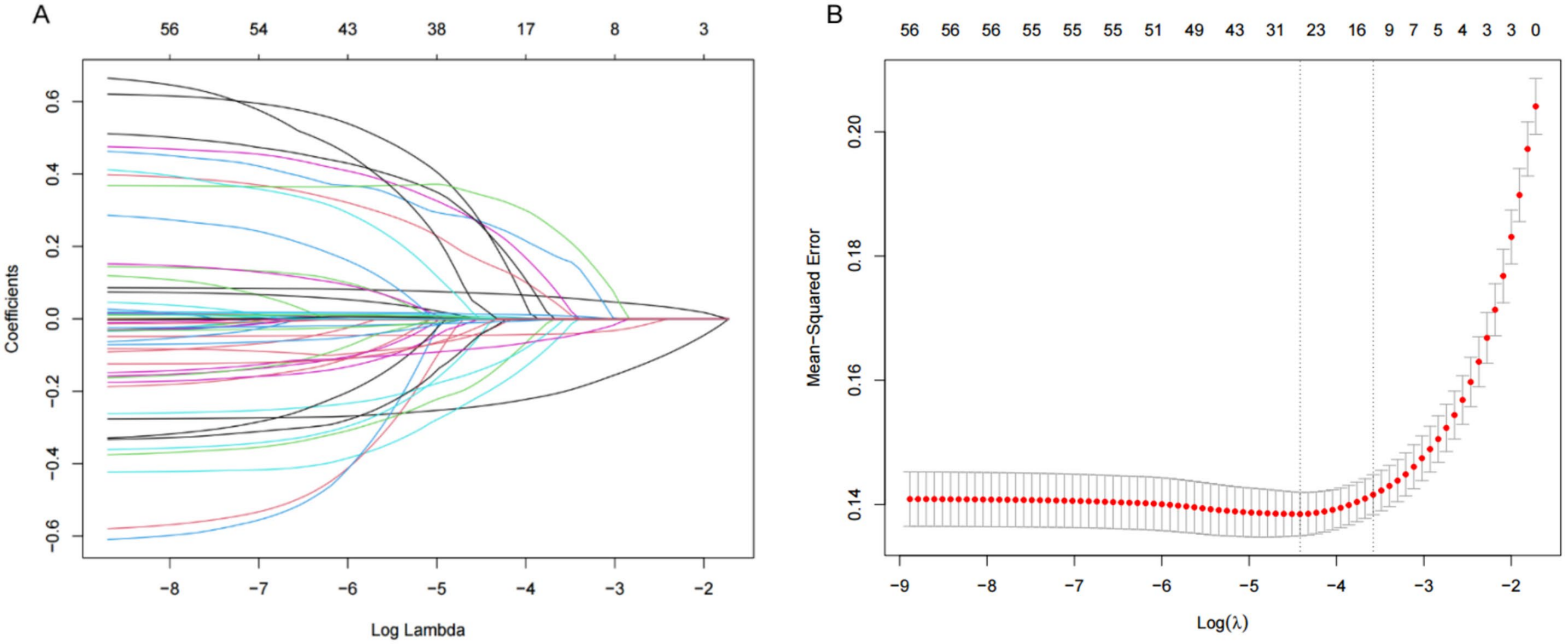
Demographic and clinical feature selection using the LASSO regression model. **(A)** According to the logarithmic (lambda) sequence, a coefficient profile was generated, and non-zero coefficients were produced by the optimal lambda. **(B)** The optimal parameter (lambda) in the LASSO model was selected via tenfold cross-validation using minimum criteria. The partial likelihood deviation (binomial deviation) curve relative to log (lambda) was plotted. A virtual vertical line at the optimal value was drawn using one SE of minimum criterion (the 1-SE criterion).

Using whether or not sarcopenia occurred in middle-aged or older adult diabetic participants as the outcome variable, all candidate predictor variables were included in LASSO regression, and the independent variable screening and cross-validation process of LASSO regression are shown in Figures 2A,B, respectively. Non-zero coefficients were selected as potential predictors of sarcopenia. In order to ensure that the model is both efficient and concise, and considering the simplicity and operability in the process of practical clinical application, the Lambda.lse value (Lambda = 0.02787465) with the cross-validation error within the range of plus or minus one standard error of the minimum error was selected as the optimal penalty coefficient in our study, at which time the model contained 13 variables, including age, gender, BMI, residence, smoking, arthritis, cognitive function, depression, social participation, ADL, PEF, DBP, and Hb. These factors were then included in the logistic regression model. It was ultimately found that, Age (*p* < 0.001), Residence (*p* = 0.003), BMI (*p* < 0.001), Cognitive function (*p* = 0.003), ADL (*p* < 0.001), DBP (*p* = 0.019), Breathing (*p* < 0.001) and Hb (*p* < 0.001) were found to be associated with the development of sarcopenia in middle-aged and older adult diabetic mellitus (Table 2).

**Table 2:** The prediction model with multivariate logistic regression.

Variables	OR	95%CI	*p*-value
Residence			0.003
Urban	Reference		
Rural	1.575	[1.163,2.133]	
ADL			<0.001
Non-disability	Reference		
Disability	1.874	[1.392,2.522]	
Cognitive function	0.927	[0.888,0.967]	<0.001
Age	1.079	[1.060,1.100]	<0.001
BMI	0.759	[0.725,0.795]	<0.001
DBP	0.984	[0.970,0.997]	0.019
PEF	0.995	[0.993,0.996]	<0.001
Hb	0.860	[0.795,0.931]	<0.001

BMI, body mass index; ADL, Activities of daily living; DBP, mean diastolic blood pressure; Hb, Hemoglobin; OR, odds ratio; CI, confidence interval.

### 3.3 Developing predictive models

**Figure 3:**
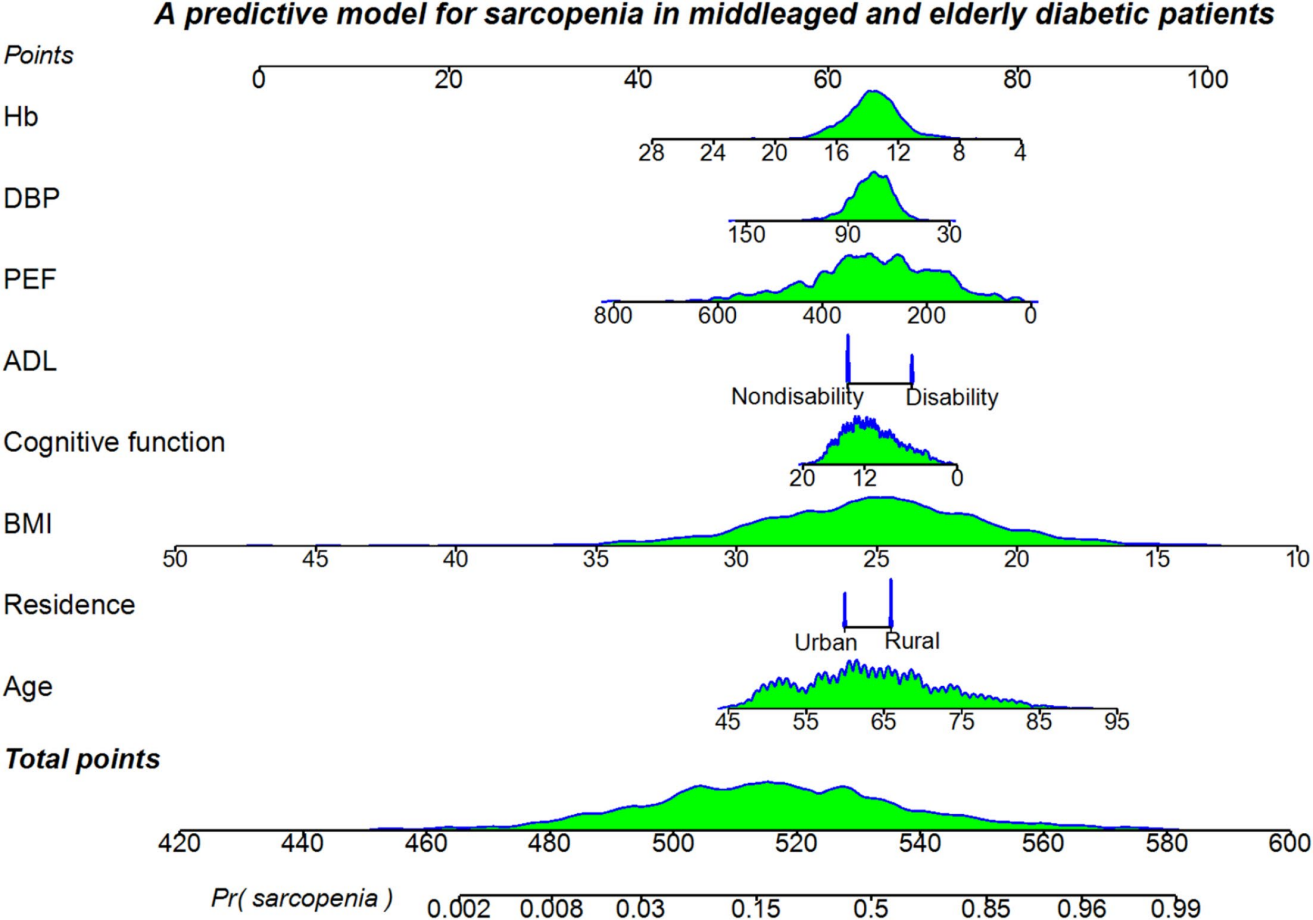
A nomogram predictive model for sarcopenia in middle-aged and older adult diabetic patients in China.

Logistic regression was used to establish the predictive model. Variance inflation factor (*VIF*) test was performed and the VIF values for all variables were in the range of 1.09 to 1.54, with *VIF* values below five. The model was well fitted with no covariance. A predictive model was presented using nomogram, which allowed for a quantitative possible predictive of sarcopenia in middle-aged and older adult diabetic patients (Figure 3). Individual scores for each predictor variable in the nomogram model ranged from 0 to 100, and total scores ranged from 420 to 600. When using the nomogram predictive model, the corresponding scores of each independent influencing factor were projected onto the first row, and then the scores of the eight influencing factors were cumulatively summed to obtain the total score, based on which the probability of the occurrence of sarcopenia in middle-aged and older adult diabetic patients was judged to be high or low.

### 3.4 Predictive model validation

**Figure 4:**
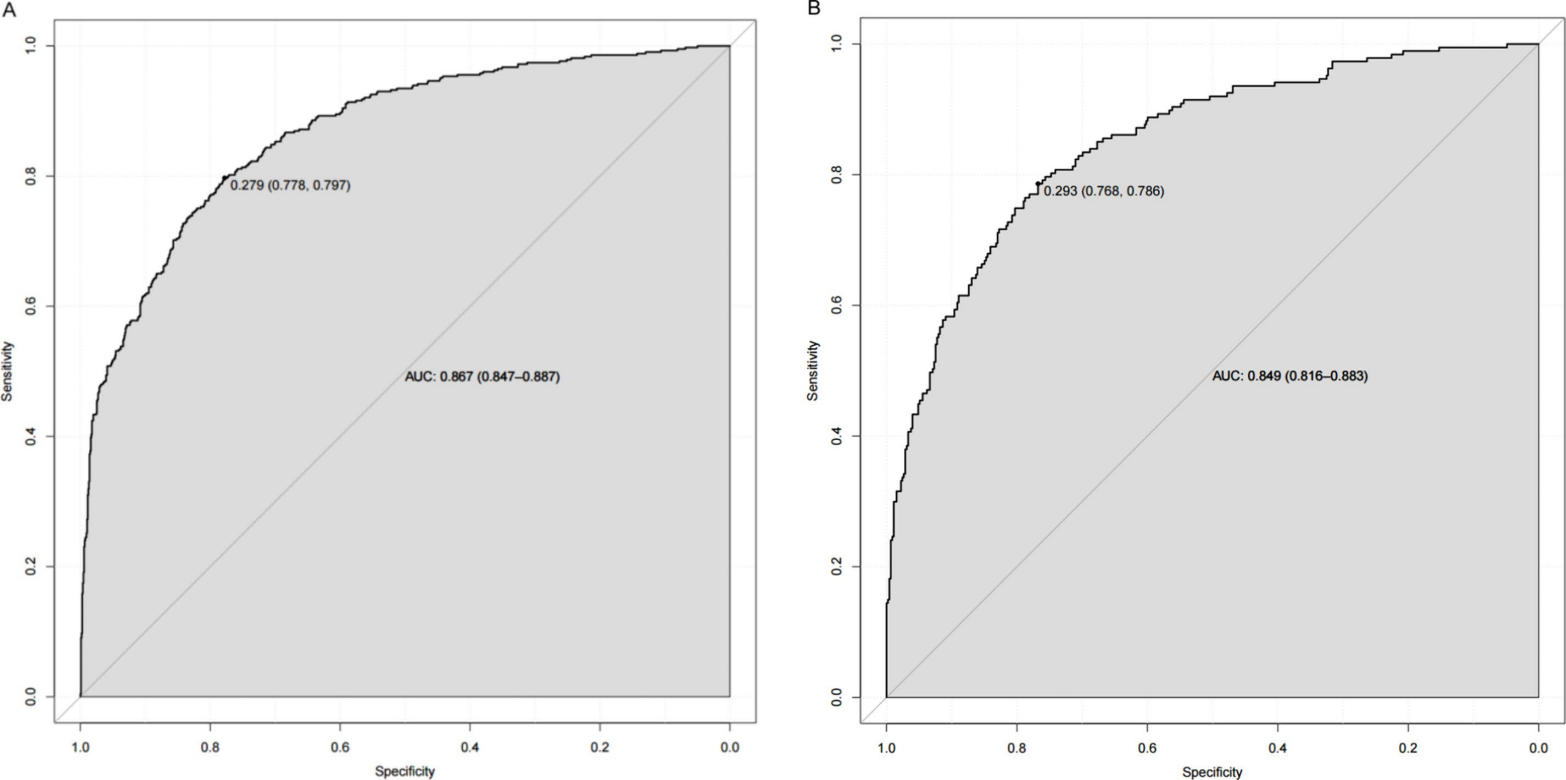
**(A)** Nomogram *ROC* curves generated from the training data set. **(B)** Nomogram *ROC* curves generated using the validation data set.

**Figure 5:**
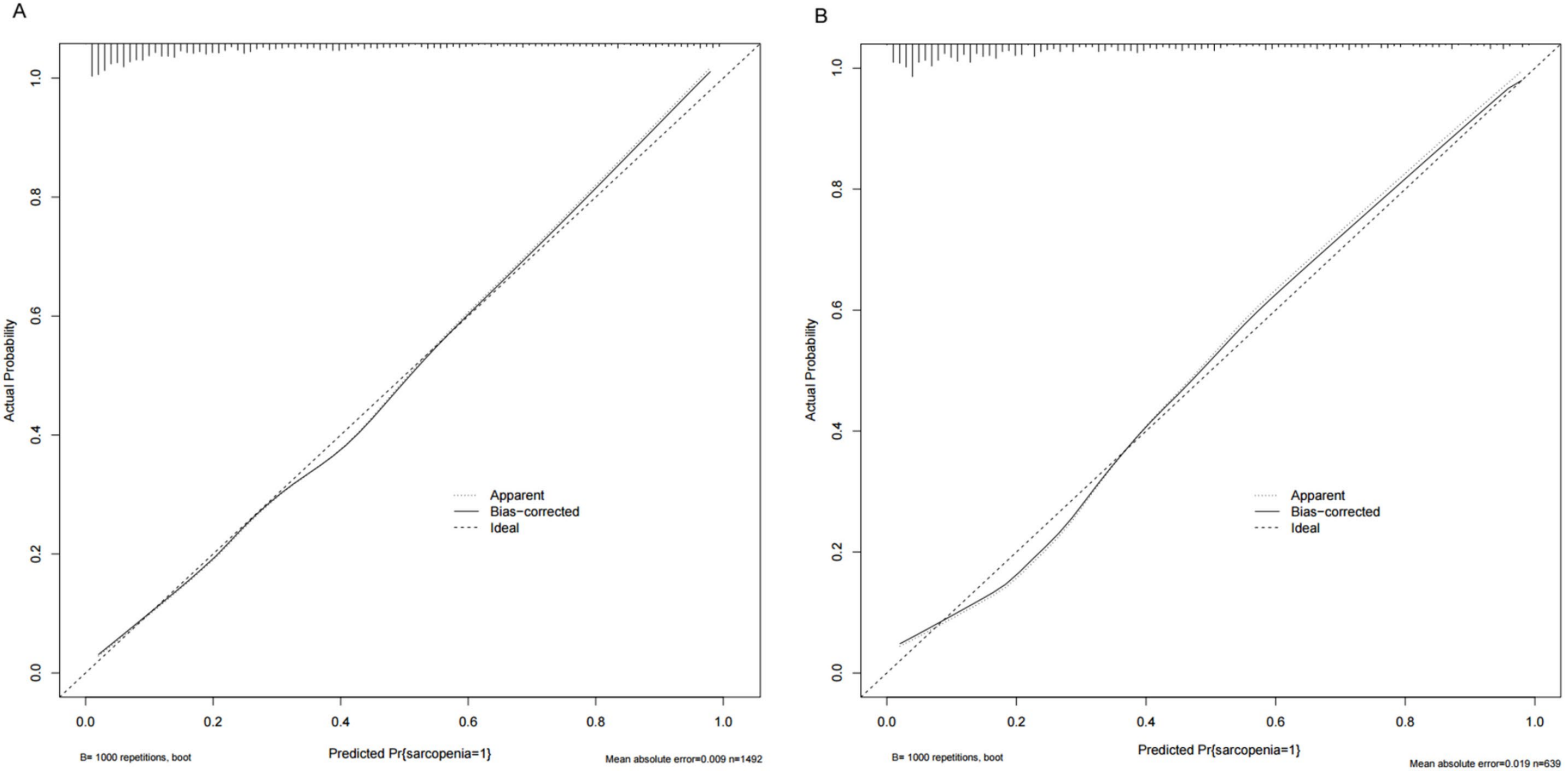
**(A)** Calibration plots for training data set. **(B)** Calibration plots for validation data set.

**Figure 6:**
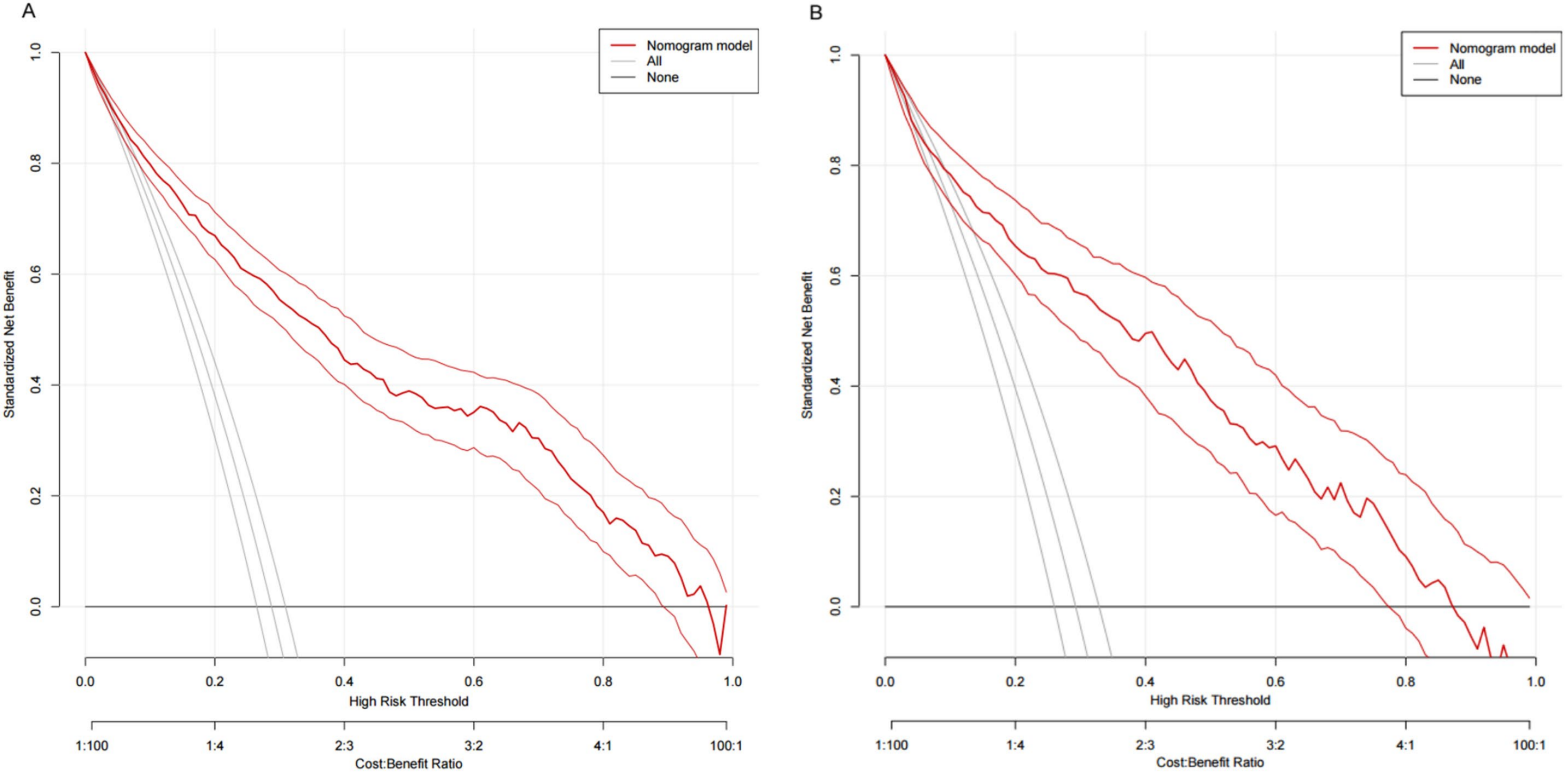
**(A)** DCA curves for training data set. **(B)** DCA curves for validation data set.

As shown in Figure 4A, the *AUC* value for the predictive model was 0.867 (95%CI: 0.847~0.887), and the optimal threshold was 0.279, sensitivity was 0.797 and specificity was 0.778. The calibration curve indicated a good model fit (χ^2^ = 13.483, *df* = 8, *p* = 0.097), *p* > 0.05, the difference was not statistically significant, suggesting that the predictive ability of the predictive model was more consistent with the actual incidence rate, and the Brier score was 0.009, indicating that the model was well calibrated (Figure 5A). The clinical validity of the model was assessed using a *DCA* curve, which showed that the training set exceeded the extremes, indicating that the nomogram predictive model provided superior net benefit and predictive accuracy (Figure 6A). Validation of the predictive model using the validation set showed an *AUC* value of 0.849 (95%CI: 0.816~0.883) for the validation set (Figure 4B), and the calibration curve indicated a good fit of the model (*χ*^2^ = 14.327, *df* = 8, *p* = 0.074) (Figure 5B). The *DCA* curves demonstrate the clinical validity of the model (Figure 6B).

## 4 Discussion

The results of this study indicated that the prevalence of sarcopenia in middle-aged and older adult diabetic patients in China was 28.9%, higher than the findings of Li et al. (25) (18.86%), and the reason for analysis was mainly related to the different ages of the included population, with the prevalence of sarcopenia gradually increasing with age, and middle-aged and older adult population as a priority group (26). Sarcopenia has been considered as the third type of complication in diabetic patients, as a new diabetic complication, presenting reversible characteristics, so the early identification of diabetic patients with the presence of sarcopenia in the high-risk group, and the prevention in advance, can delay or even reverse the occurrence of sarcopenia.

In this study, age was found to be a risk factor for the development of sarcopenia in diabetic mellitus. Older adult patients are already a high prevalence group for sarcopenia, and the prevalence of sarcopenia has been as high as 6.8% to 18.5% in the Chinese community’s older adult population, and even higher in the middle-aged and older adult population with associated chronic diseases (27). The decline in muscle mass and muscle strength with age is greater in diabetic patients than in non-diabetic patients (5, 28, 29). After the age of 50 years, muscle mass decreases by approximately 1 to 2% per year, accompanied by a progressive loss of muscle mass, strength and function due to a decrease in the number and size of type II muscle fibres (30, 31). Therefore, muscle mass in the older adult diabetic population needs to be taken care of.

The results of the present study showed that lower BMI increased the risk of developing sarcopenia, similar to the findings of Chen et al. (32). Decrease in BMI was associated with low muscle mass, and lower BMI was considered as a dietary tendency to choose a lower protein content for intake. However, Dai et al. (33) found that the risk of developing sarcopenia was about 6.12 times higher when the BMI was ≥30 kg/m^2^. Middle-aged and older adult diabetic patients with high body fat may be more likely to develop sarcopenia, considered as infiltration of adipose tissue in muscle and bone tissue stimulates sarcopenia (34). Adipose tissue can also cause chronic inflammation, leading to adipose-secreted cytokines that inhibit muscle protein synthesis, osteoblast differentiation and production, resulting in abnormal bone tissue and muscle function (35). Therefore, in addition to diabetes patients with low BMI, obese individuals should also be the focus of attention when evaluating for community sarcopenia groups.

The results of this study also showed that decreased Hb is a risk factor for sarcopenia, and decreased Hb responds to conditions such as anaemia and malnutrition in the organism (36, 37). In diabetic mellitus patients, the normal energy intake of the organism is affected due to long-term dietary control and medication. If energy intake is low and cannot match the level of energy expenditure, it leads to weight loss and muscle mass loss in the older adult, and loss of muscle mass and function is the main feature of sarcopenia (5). As a result, a diabetic diet can be customized based on the patient’s BMI and Hb test results. Maintaining a healthy nutritional status is crucial for blood glucose control and preventing sarcopenia.

The results of the study showed that the prevalence of sarcopenia was higher in middle-aged and older adult diabetic patients living in rural areas compared to those living in urban areas. For middle-aged and older adult diabetic patients living in urban areas, early diagnosis and prevention of the illness are simpler, and information is easier to obtain (38). At the same time, some activities and exercise venues and equipment are more available in the city. Therefore, in the future, middle-aged and older adult people living in rural areas should pay more attention to muscle exercise and DM health education.

Our study revealed that the risk of sarcopenia increases with poorer cognitive function. DM patients are prone to cognitive dysfunction due to thickening of the basement membrane of the brain and increased permeability of the blood–brain barrier, where proteins and other plasma constituents penetrate into the perivascular compartments and damage neurons (39, 40). In individuals with diabetes, cognitive impairment may be a contributing factor to a decrease in physical activity duration and an increase in the frequency and length of periods of inactivity and lying down. Secondly, low cognitive function can lead to difficulties with dietary intake and reduced eating, and diabetics themselves require long-term dietary control due to disease factors, all of which accelerate sarcopenia. Notably, hyperglycaemia may also lead to oxidative stress and inflammatory responses, which are common mechanisms for both cognitive impairment and sarcopenia (28). Focusing on muscle function is therefore much more crucial for people with DM who also have cognitive problems.

The results of this study showed that disability is a risk factor for sarcopenia in diabetic patients. Chronic disruptions in glucose metabolism can result in a number of consequences, including cardiovascular disease, nephropathy, and neuropathy. These conditions can make it more difficult for the patient to take care of themselves and raise the risk of incapacitation (41). Disability most directly leads to decreased mobility and increased prevalence of sarcopenia. Sarcopenia should therefore be considered while screening for disabilities.

Peak expiratory flow (PEF) is a simple screening tool for lung function and is defined as the instantaneous velocity at the fastest expiratory flow during exertion spirometry, reflecting the strength of the respiratory muscles (42). In this study, PEF was found to be a risk factor for the development of sarcopenia in middle-aged and older adult DM patients. The hyperglycaemic state of the body in diabetic patients affects lung physiology, inflammation and bacterial infections, which may lead to a loss of respiratory muscle mass and strength, and/or a decrease in lung function, which is known as sarcopenia (43). According to the 2010 consensus of the European Working Group on Sarcopenia in the older adult, PEF is determined by respiratory muscle strength in people without lung disease (5), and it can be used as an indicator of respiratory sarcopenia (44). Consequently, middle-aged and older diabetes patients with pulmonary dysfunction must actively manage their blood glucose levels. Lip-contraction belly breathing training is one way to strengthen the respiratory muscles and lessen respiratory sarcopenia.

Also, this study found that low diastolic blood pressure is strongly associated with the development of sarcopenia. Diastolic blood pressure is the pressure generated by the elastic retraction of arterial blood vessels when the heart is in diastole, and low diastolic blood pressure may indicate that the heart is not pumping enough blood to the body during diastole, which may also affect the supply of nutrients to the muscles (44). Low diastolic blood pressure may be a sign of declining fitness in the older adult, which is closely associated with sarcopenia. Therefore, in the future, patients with DM combined with hypertension should be concerned about muscle loss in addition to cardiac, cerebral, and renal complications.

The nomogram prediction model constructed in our study has a good prediction performance. First, the area under the ROC curve of the model is 0.867, which is greater than 0.800, indicating that the model has a good degree of discrimination. Second, the calibration curve of the model is close to the standard curve (45-degree line), indicating a high degree of agreement between the predicted probabilities and the observations. Finally, the DCA curve is within the probability threshold interval and above the reference line, indicating that the model has some clinical application value. Our study compares with the study by Zhang et al. (45) on sarcopenia in the older adult, where BMI and DBP were common predictors in both studies, but the *AUC* in our study was 0.867 (95%CI: 0.847~0.887) much higher than the latter (0.77, 95%CI: 0.75~0.79). And the results of the present study were more applicable to DM population. As shown in the nomogram predictive model, BMI was relatively more important, followed by PEF, age, Hb, DBP, cognitive function, ADL, and residence. The nomogram predictive model is simple and easy to use and the eight predictors are easily accessible. Community workers can assign values to each predictor and calculate the total score, and differentiate between high- and low-risk groups according to the optimal threshold value of 0.279, which not only reduces the computational burden of the users, but also saves the time of the assessment effectively.

The predictive model we constructed had good discrimination and accuracy, and the results of internal validation were found to be a valuable tool for assessing sarcopenia in DM patients. However, this study also has some limitations. Firstly, only data from 2015 in the CHARLS database were selected for this study, which was retrospective. Second, this study was only internally validated without external validation, and the study population was from China, which limits its generalization. Finally, the survey method of this study is self-report, the results of the questions will be affected by the subjective consciousness of the patients, and this study selected the middle-aged and older adult population, some of them will have memory bias, and the accuracy of the results may be affected to some extent. Large-sample, multi-center studies should be carried out in the future to investigate causality and validate the results.

## 5 Conclusion

Our study constructed a risk predictive model for sarcopenia in middle-aged and older adult DM patients with good predictive efficacy, which screened 8 predictors, including age, gender, residence, PEF, Hb, DBP, cognitive function, and ADL. The predictive model was built by combining the above 8 independent risk factors for sarcopenia in diabetic patients, which transformed the complex equation into a visual model. This novel screening tool is accurate, specific, and cost-effective, highlighting its potential value in clinical applications.

## Data Availability

The original contributions presented in the study are included in the article/Supplementary material, further inquiries can be directed to the corresponding author.

## References

[1] Khan MAB Hashim MJ King JK Govender RD Mustafa H Al KJ. Epidemiology of type 2 diabetes – global burden of disease and forecasted frends. J Epidemiol Glob Health. (2020) 10:107–11. doi: 10.2991/jegh.k.191028.001, PMID: 32175717 PMC7310804

[2] Sun H Saeedi P Karuranga S Pinkepank M Ogurtsova K Duncan BB. IDF diabetes atlas: global, regional and country-level diabetes prevalence estimates for 2021 and projections for 2045. Diabetes Res Clin Pract. (2022) 183:109119. doi: 10.1016/j.diabres.2021.109119, PMID: 34879977 PMC11057359

[3] Saeedi P Petersohn I Salpea P Malanda B Karuranga S Unwin N. Global and regional diabetes prevalence estimates for 2019 and projections for 2030 and 2045: results from the international diabetes federation diabetes atlas, 9th edition. Diabetes Res Clin Pract. (2019) 157:107843. doi: 10.1016/j.diabres.2019.107843 31518657

[4] Yan Y Wu T Zhang M Li C Liu Q Li F. Prevalence, awareness and control of type 2 diabetes mellitus and risk factors in Chinese elderly population. BMC Public Health. (2022) 22:1382. doi: 10.1186/s12889-022-13759-9, PMID: 35854279 PMC9295461

[5] Cruz-Jentoft AJ Baeyens JP Bauer JM Boirie Y Cederholm T Landi F. Sarcopenia: European consensus on definition and diagnosis: report of the European working group on sarcopenia in older people. Age Ageing. (2010) 39:412–23. doi: 10.1093/ageing/afq034, PMID: 20392703 PMC2886201

[6] Wang T. Searching for the link between inflammaging and sarcopenia. Ageing Res Rev. (2022) 77:101611. doi: 10.1016/j.arr.2022.101611, PMID: 35307560

[7] Beaudart C Demonceau C Reginster JY Locquet M Cesari M Cruz Jentoft AJ. Sarcopenia and health-related quality of life: a systematic review and meta-analysis. J Cachexia Sarcopenia Muscle. (2023) 14:1228–43. doi: 10.1002/jcsm.13243, PMID: 37139947 PMC10235892

[8] Mesinovic J Fyfe JJ Talevski J Wheeler MJ Leung GKW George ES. Type 2 diabetes mellitus and sarcopenia as comorbid chronic diseases in older adults: established and emerging treatments and therapies. Diabetes Metab J. (2023) 47:719–42. doi: 10.4093/dmj.2023.0112, PMID: 37709502 PMC10695715

[9] Wang T Feng X Zhou J Gong H Xia S Wei Q. Type 2 diabetes mellitus is associated with increased risks of sarcopenia and pre-sarcopenia in Chinese elderly. Sci Rep. (2016) 6:38937. doi: 10.1038/srep38937, PMID: 27958337 PMC5153616

[10] Yeung SSY Reijnierse EM Pham VK Trappenburg MC Lim WK Meskers CGM. Sarcopenia and its association with falls and fractures in older adults: a systematic review and meta-analysis. J Cachexia Sarcopenia Muscle. (2019) 10:485–500. doi: 10.1002/jcsm.12411, PMID: 30993881 PMC6596401

[11] Qiao YS Chai YH Gong HJ Zhuldyz Z Stehouwer CDA Zhou JB. The association between diabetes mellitus and risk of sarcopenia: accumulated evidences from observational studies. Front Endocrinol. (2021) 12:782391. doi: 10.3389/fendo.2021.782391, PMID: 35002965 PMC8734040

[12] Li Q Cheng H Cen W Yang T Tao S. Development and validation of a predictive model for the risk of sarcopenia in the older adults in China. Eur J Med Res. (2024) 29:278. doi: 10.1186/s40001-024-01873-w, PMID: 38725036 PMC11084063

[13] American Diabetes Association Professional Practice Committee. Classification and Diagnosis of Diabetes: Standards of medical care in diabetes-2022. Diabetes Care. (2022) 45:S17–s38. doi: 10.2337/dc22-S002, PMID: 34964875

[14] Chen LK Woo J Assantachai P Auyeung TW Chou MY Iijima K. Asian working group for sarcopenia: 2019 consensus update on sarcopenia diagnosis and treatment. J Am Med Dir Assoc. (2020) 21:300–7.e2. doi: 10.1016/j.jamda.2019.12.012, PMID: 32033882

[15] Yang M Hu X Wang H Zhang L Hao Q Dong B. Sarcopenia predicts readmission and mortality in elderly patients in acute care wards: a prospective study. J Cachexia Sarcopenia Muscle. (2017) 8:251–8. doi: 10.1002/jcsm.12163 27896949 PMC5377397

[16] Wen X Wang M Jiang CM Zhang YM. Anthropometric equation for estimation of appendicular skeletal muscle mass in Chinese adults. Asia Pac J Clin Nutr. (2011) 20:551–6. 22094840

[17] Wu X Li X Xu M Zhang Z He L Li Y. Sarcopenia prevalence and associated factors among older Chinese population: findings from the China health and retirement longitudinal study. PLoS One. (2021) 16:e247617. doi: 10.1371/journal.pone.0247617, PMID: 33661964 PMC7932529

[18] Jin X He J Liang Y Sun X Yan S Wu Y. Associations between household solid fuel use and activities of daily living trajectories: a nationwide longitudinal study of middle and older adults in China. Environ Int. (2022) 170:107605. doi: 10.1016/j.envint.2022.107605 36323064

[19] Yuan M Qin F Zhou Z Fang Y. Gender-specific effects of adverse childhood experiences on incidence of activities of daily life disability in middle-age and elderly Chinese population. Child Abuse Negl. (2021) 117:105079. doi: 10.1016/j.chiabu.2021.105079, PMID: 33945896

[20] Ning H Zhang H Xie Z Jiang W Xie S. Relationship of hearing impairment, social participation and depressive symptoms to the incidence of frailty in a community cohort. J Am Geriatr Soc. (2023) 71:1167–76. doi: 10.1111/jgs.18164, PMID: 36504135

[21] Feng Z Cramm JM Jin C Twisk J Nieboer AP. The longitudinal relationship between income and social participation among Chinese older people. SSM-Popul Health. (2020) 11:100636. doi: 10.1016/j.ssmph.2020.100636, PMID: 32802932 PMC7419328

[22] Boey KW. Cross-validation of a short form of the CES-D in Chinese elderly. Int J Geriatr Psychiatry. (1999) 14:608–17. doi: 10.1002/(SICI)1099-1166(199908)14:8<608::AID-GPS991>3.0.CO;2-Z 10489651

[23] Zhang W Ding Z Peng Y Wang H Sun Y Ke H. LUTS/BPH increases the risk of depressive symptoms among elderly adults: a 5-year longitudinal evidence from CHARLS. J Affect Disord. (2024) 367:210–8. doi: 10.1016/j.jad.2024.08.205, PMID: 39233239

[24] Lei X Liu H. Gender difference in the impact of retirement on cognitive abilities: evidence from urban China. J Comp Econ. (2018) 46:1425–46. doi: 10.1016/j.jce.2018.01.005

[25] Li R Lin S Tu J Chen Y Cheng B Mo X. Establishment and evaluation of a novel practical tool for the diagnosis of pre-sarcopenia in young people with diabetes mellitus. J Transl Med. (2023) 21:393. doi: 10.1186/s12967-023-04261-w, PMID: 37330547 PMC10276365

[26] Koo BK Roh E Yang YS Moon MK. Difference between old and young adults in contribution of β-cell function and sarcopenia in developing diabetes mellitus. J Diabetes Investig. (2015) 7:233–40. doi: 10.1111/jdi.12392, PMID: 27042276 PMC4773679

[27] Yang LJ Wu GH Yang YL Wu YH Zhang L Wang MH. Nutrition, physical exercise, and the prevalence of sarcopenia in elderly residents in nursing homes in China. Med Sci Monit. (2019) 25:4390–9. doi: 10.12659/MSM.914031, PMID: 31189870 PMC6587647

[28] Shatila H Ghazal N Bukshaisha G Al-Zeyara S Khoury CFE Bassil M. Risk and determinants of sarcopenia in people with diabetes: a case-control study from Qatar biobank cohort. BMC Endocr Disord. (2024) 24:205. doi: 10.1186/s12902-024-01722-1, PMID: 39350192 PMC11440684

[29] Chen H Huang X Dong M Wen S Zhou L Yuan X. The association between sarcopenia and diabetes: from pathophysiology mechanism to therapeutic strategy. Diabetes Metab Syndr Obes. (2023) 16:1541–54. doi: 10.2147/DMSO.S410834, PMID: 37275941 PMC10239259

[30] Sieber CC. Malnutrition and sarcopenia. Aging Clin Exp Res. (2019) 31:793–8. doi: 10.1007/s40520-019-01170-1 31148100

[31] Mathewson SL Azevedo PS Gordon AL Phillips BE Greig CA. Overcoming protein-energy malnutrition in older adults in the residential care setting: a narrative review of causes and interventions. Ageing Res Rev. (2021) 70:101401. doi: 10.1016/j.arr.2021.101401 34237434

[32] Chen F Xu S Wang Y Chen F Cao L Liu T. Risk factors for sarcopenia in the elderly with type 2 diabetes mellitus and the effect of metformin. J Diabetes Res. (2020) 2020:3950404. doi: 10.1155/2020/3950404, PMID: 33083494 PMC7563046

[33] Dai S Shu D Meng F Chen Y Wang J Liu X. Higher risk of sarcopenia in older adults with type 2 diabetes: NHANES 1999-2018. Obes Facts. (2023) 16:237–48. doi: 10.1159/000530241, PMID: 37011596 PMC10826600

[34] Merchant RA Seetharaman S Au L Wong MWK Wong BLL Tan LF. Relationship of fat mass index and fat free mass index with body mass index and association with function, cognition and sarcopenia in pre-frail older adults. Front Endocrinol (Lausanne). (2021) 12:765415. doi: 10.3389/fendo.2021.765415, PMID: 35002957 PMC8741276

[35] Walsh JS Vilaca T. Obesity, type 2 diabetes and bone in adults. Calcified Tissue Int. (2017) 100:528–35. doi: 10.1007/s00223-016-0229-0, PMID: 28280846 PMC5394147

[36] Avila JC Samper-Ternent R Wong R. Malnutrition risk among older Mexican adults in the Mexican health and aging study. Nutrients. (2021) 13:1615. doi: 10.3390/nu13051615, PMID: 34065807 PMC8151238

[37] Zhang Z Pereira SL Luo M Matheson EM. Evaluation of blood biomarkers associated with risk of malnutrition in older adults: a systematic review and meta-analysis. Nutrients. (2017) 9:829. doi: 10.3390/nu9080829, PMID: 28771192 PMC5579622

[38] Sauliune S Mesceriakova-Veliuliene O Kalediene R. Inequalities in life expectancy by place of residence and its changes in Lithuania during 1990-2018. Eur J Pub Health. (2024) 30:30. doi: 10.1093/eurpub/ckaa165.450

[39] Han F Kong X Lv W Li S Sun Y Wu Y. Association of diabetes mellitus with gait and falls in community-dwelling older adults: serial mediation of vision and cognition. Arch Gerontol Geriat. (2022) 104:104827. doi: 10.1016/j.archger.2022.104827, PMID: 36191493

[40] Callisaya ML Beare R Moran C Phan T Wang W Srikanth VK. Type 2 diabetes mellitus, brain atrophy and cognitive decline in older people: a longitudinal study. Diabetologia. (2018) 62:448–58. doi: 10.1007/s00125-018-4778-9, PMID: 30547230

[41] Joshi SA Patel VD Eapen C Hariharan K. Proportion and distribution of upper extremity musculoskeletal disorders and its association with disability in type 2 diabetes mellitus. J Hand Ther. (2021) 35:597–604. doi: 10.1016/j.jht.2021.04.013, PMID: 34016518

[42] Fragoso CAV Gill TM. Respiratory impairment and the aging lung: a novel paradigm for assessing pulmonary function. J Gerontol A Biol Sci Med Sci. (2012) 67:264–75. doi: 10.1093/gerona/glr198, PMID: 22138206 PMC3297762

[43] Nagano A Wakabayashi H Maeda K Kokura Y Miyazaki S Mori T. Respiratory sarcopenia and sarcopenic respiratory disability: concepts, diagnosis, and treatment. J Nutr Health Aging. (2021) 25:507–15. doi: 10.1007/s12603-021-1587-5, PMID: 33786569 PMC7799157

[44] Kera T Kawai H Hirano H Kojima M Watanabe Y Motokawa K. Definition of respiratory sarcopenia with peak expiratory flow rate. J Am Med Dir Assoc. (2019) 20:1021–5. doi: 10.1016/j.jamda.2018.12.013, PMID: 30737167

[45] Zhang XZ Xie WQ Chen L Xu GD Wu L Li YS. Blood flow restriction training for the intervention of sarcopenia: current stage and future perspective. Front Med (Lausanne). (2022) 9:894996. doi: 10.3389/fmed.2022.89499 35770017 PMC9234289

