# Supplementary figures and images for: Development and validation of a predictive model for the risk of possible sarcopenia in middle-aged and older adult diabetes mellitus in China
Source: Front Public Health. 2025 Apr 3;13:1521736. doi: 10.3389/fpubh.2025.1521736 (PMC12003298; doi:10.3389/fpubh.2025.1521736)

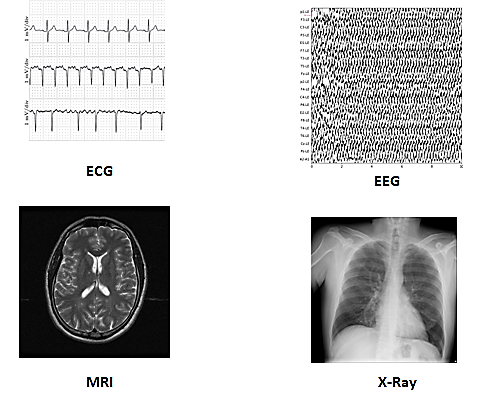

Supplement: Supplementary file 1 [file Image_1.PNG]

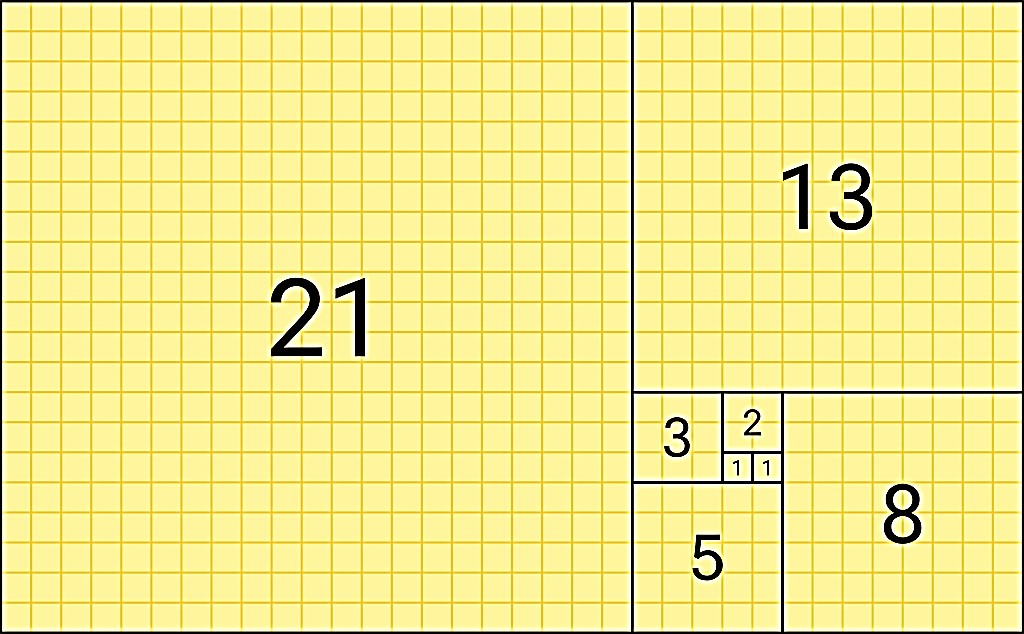

Supplement: Supplementary file 2 [file Image_2.PNG]

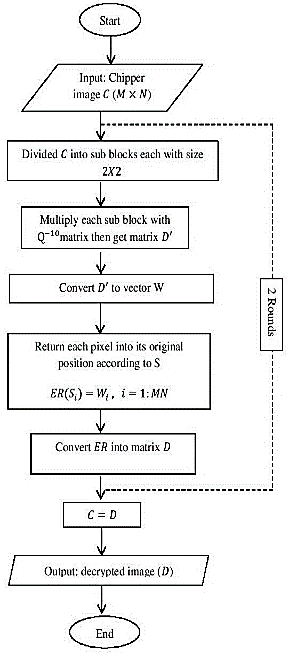

Supplement: Supplementary file 3 [file Image_3.PNG]

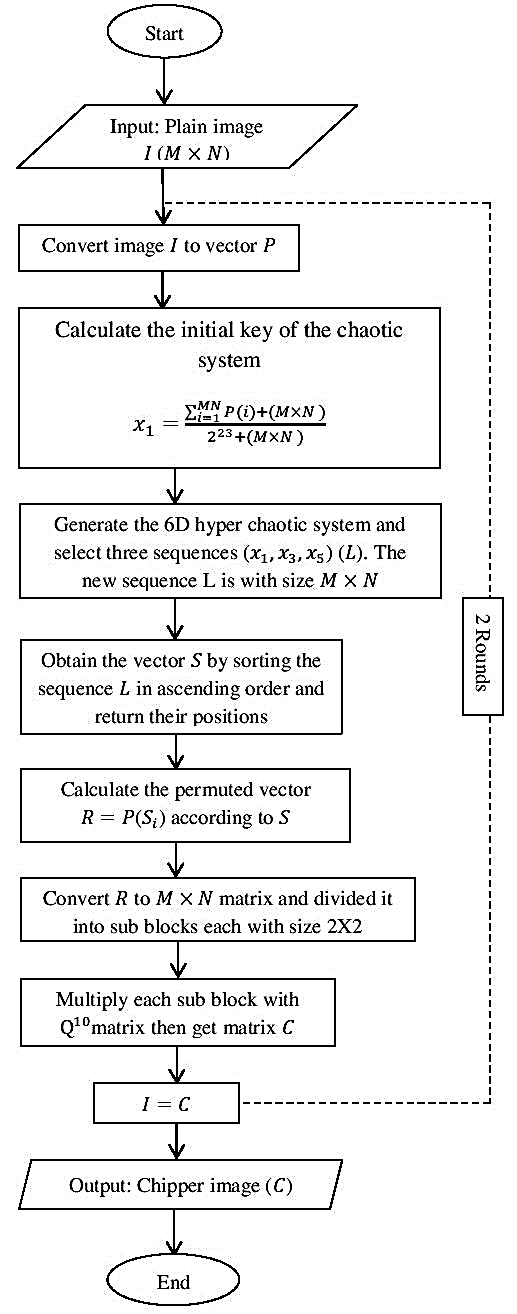

Supplement: Supplementary file 4 [file Image_4.PNG]

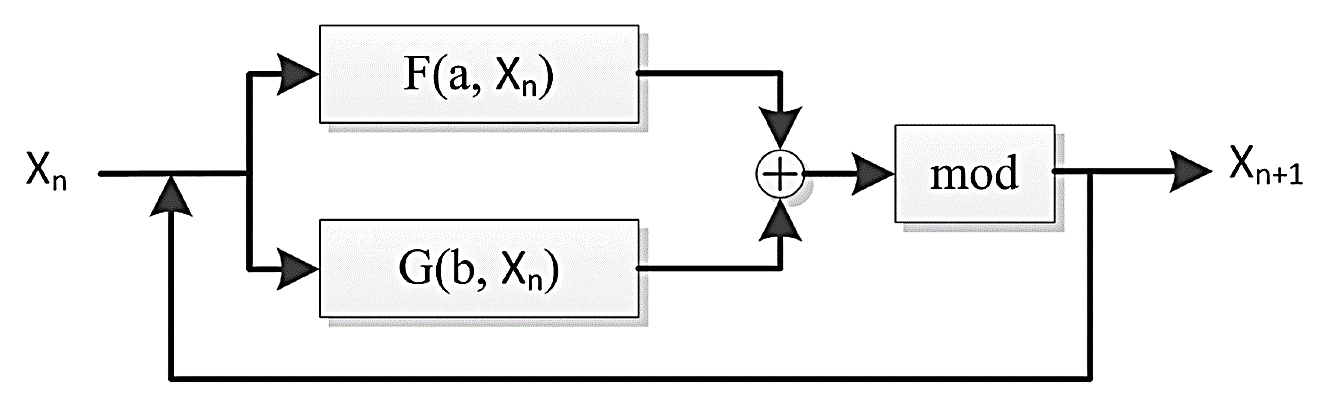

Supplement: Supplementary file 5 [file Image_5.PNG]

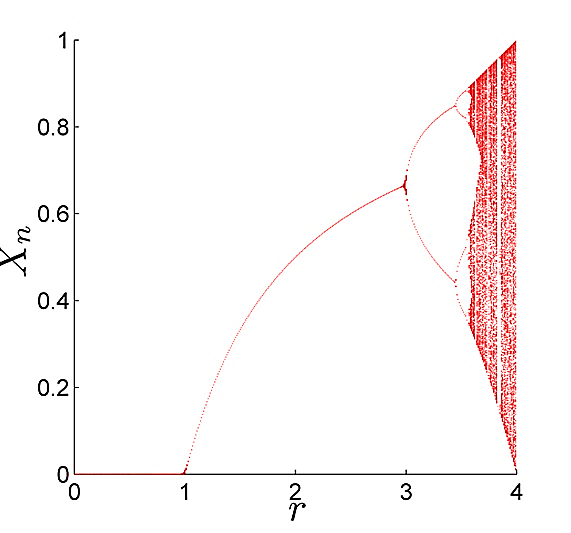

Supplement: Supplementary file 6 [file Image_6.PNG]

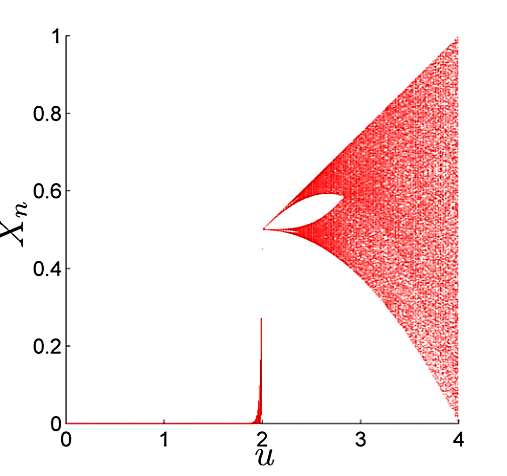

Supplement: Supplementary file 7 [file Image_7.PNG]

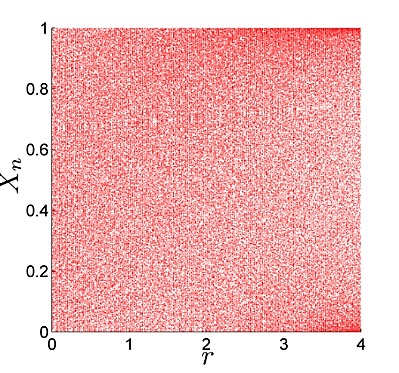

Supplement: Supplementary file 8 [file Image_8.PNG]

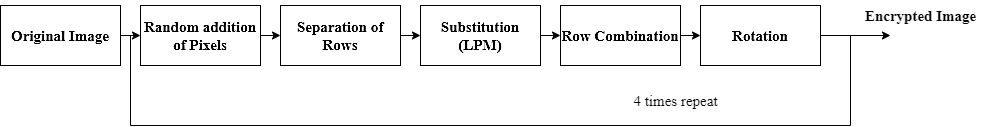

Supplement: Supplementary file 9 [file Image_9.PNG]

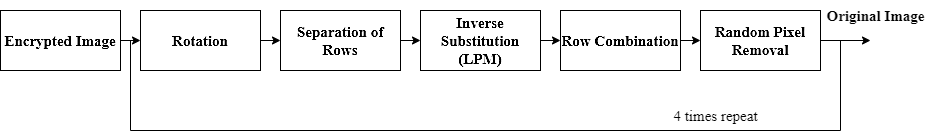

Supplement: Supplementary file 10 [file Image_10.PNG]

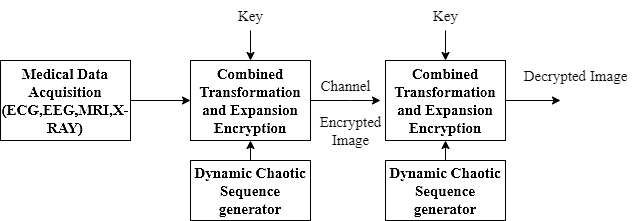

Supplement: Supplementary file 11 [file Image_11.PNG]

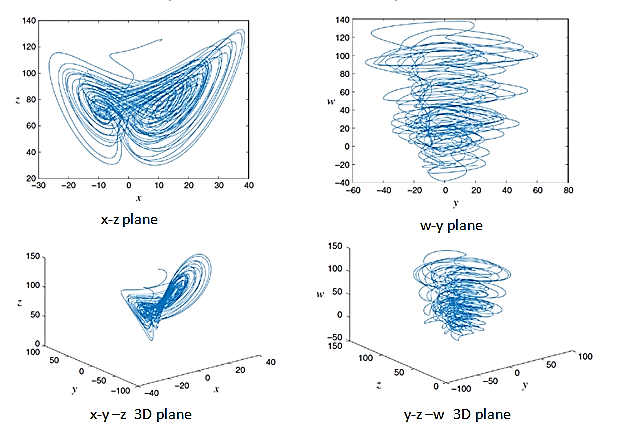

Supplement: Supplementary file 12 [file Image_12.PNG]

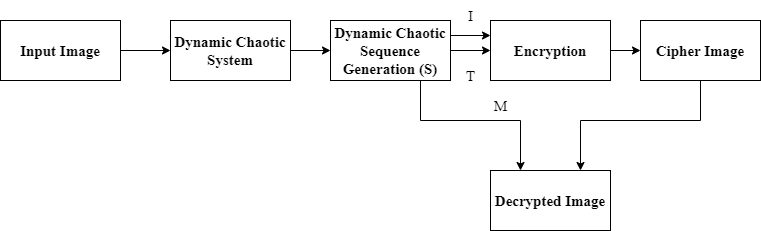

Supplement: Supplementary file 13 [file Image_13.PNG]

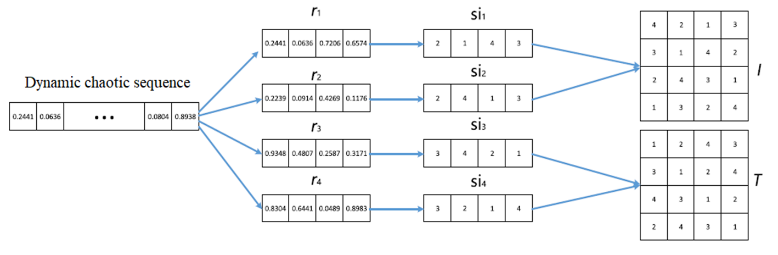

Supplement: Supplementary file 14 [file Image_14.PNG]

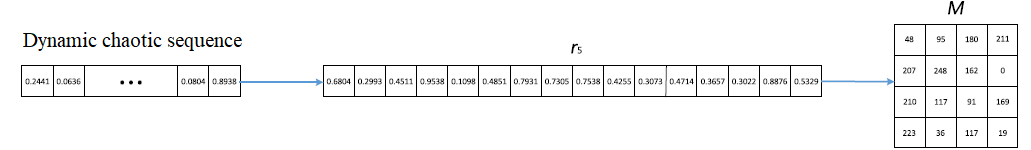

Supplement: Supplementary file 15 [file Image_15.PNG]
